# Epidemiology of low-proteinuric chronic kidney disease in renal clinics

**DOI:** 10.1371/journal.pone.0172241

**Published:** 2017-02-17

**Authors:** Luca De Nicola, Michele Provenzano, Paolo Chiodini, Silvio Borrelli, Luigi Russo, Antonio Bellasi, Domenico Santoro, Giuseppe Conte, Roberto Minutolo

**Affiliations:** 1 Nephrology Unit at Second University of Naples, Naples, Italy; 2 Medical Statistics Unit at Second University of Naples, Naples, Italy; 3 Nephrology Unit at University Federico II in Naples, Naples, Italy; 4 Nephrology Unit at Sant’Anna Hospital in Como, Como, Italy; 5 Nephrology Unit at University of Messina, Messina, Italy; Istituto Di Ricerche Farmacologiche Mario Negri, ITALY

## Abstract

CKD patients with low-grade proteinuria (LP) are common in nephrology clinics. However, prevalence, characteristics, and the competing risks of ESRD and death as the specific determinants, are still unknown. We analyzed epidemiological features of LP status in a prospective cohort of 2,340 patients with CKD stage III-V referred from ≥6 months in 40 nephrology clinics in Italy. LP status was defined as proteinuria <0.5 g/24h according to current KDIGO guidelines. Patients with higher proteinuria constituted the control group (CON). LP patients were 54.5% of the whole cohort. As compared to CON, LP were older (70.0±12.1 vs 65.4±14.1 y), and less likely to be male (55.8 vs 62.0%) and diabetic (27.6 vs 34.1%), and had hypertension as the most common cause of CKD (39.8%). They had higher eGFR (34.8±13.5 vs 26.8±13.2 mL/min/1.73m^2^) and hemoglobin (12.7±1.7 vs 12.3±1.7 g/dL), while systolic blood pressure (137±18 vs 140±18 mmHg) and serum phosphorus (3.7±0.8 vs 3.9±0.8 mg/dL) were lower [P<0.001 for all comparisons]. Over a median follow-up of 48 months, an inverse relative risk of ESRD and death was observed in LP (death>>ESRD; P = 0.002) versus CON (ESRD>>death; P<0.0001). Modifiable risk factors were also different in LP, with smoking, lower hemoglobin, and proteinuria being associated with higher mortality risk while lower BMI and higher phosphorus predicting ESRD at multivariable Cox analyses [P<0.05 for all]. Therefore, in nephrology clinics, LP patients are the majority and show distinctive basal features. More important, they are more exposed to death than ESRD and do present specific modifiable determinants of either outcome; indeed, in LP, while smoking plays a role for mortality, lower BMI and higher phosphorus levels -even if in the normal range- are predictors of ESRD. These data support the need to further study the low proteinuric CKD population to guide management.

## Introduction

In current nephrology practice, a limited number of nephrologists must cope with the growing population of non-dialysis chronic kidney disease (ND-CKD) patients characterized by advanced disease and higher burden of comorbidities requiring watchful and time-consuming care [1]. Therefore, optimizing risk stratification in this clinical setting becomes of paramount importance because it allows to properly individualize clinical management in terms of monitoring as treatment.

Previous analyses have demonstrated that in ND-CKD proteinuria is a major risk factor of cardiorenal outcome besides and beyond age, CKD stage and type of primary renal disease [2–9]. Indeed, the most recent Kidney Disease: Improving Global Outcomes (KDIGO) clinical practice guidelines for the evaluation and management of CKD have identified the proteinuria level of 0.5 g/24h as a meaningful threshold to define CKD severity in general and high-risk populations [10]. However, outcome and risk factors of CKD patients with low-grade proteinuria, that is, less than 0.5 g/24h, that are regularly followed in renal clinics are still undefined. Interest on this issue is remarkable because cross-sectional studies, in the general population as in different clinical settings, have shown that low-proteinuric patients are common [11–16]. In particular, a recent cross-sectional analysis of basal features of ND-CKD patients under nephrology care has evidenced that these patients are the majority of CKD population with low eGFR [16]. Nevertheless, no prognostic information for the low-proteinuric condition in nephrology clinics, as for other clinical settings, have been provided so far.

To fill this important gap of knowledge, we studied a large population of low-proteinuric patients with ND-CKD stage III-V under stable nephrology care to evaluate their epidemiologic features, and prognosis in terms of risks of end-stage renal disease (ESRD) and all-cause mortality as the specific determinants of either outcome. Patients with proteinuria higher than 0.5 g/24h constituted the control group.

Noteworthy, survival analyses accounted for the underlying renal disease, that *per se* mainly influences the degree of proteinuria and outcome as well, and for the competing nature of the risk of ESRD and mortality [17]. This latter point is critical; competing risk analysis in fact allows to estimate the “full” effect of risk factors for CKD progression because many ND-CKD patients do not reach ESRD as they die before [2–9]. Results provide useful information to identify potential therapeutic targets and design future trials in low-proteinuric CKD.

## Methods

### Study design

**Fig 1:**
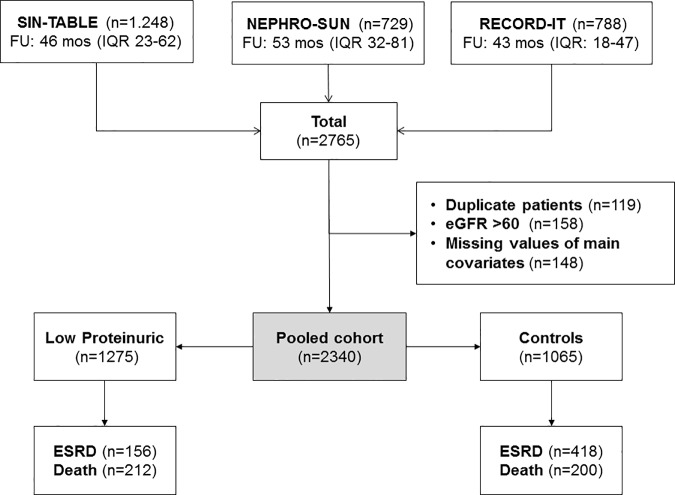
Flow chart of the study.

This is an observational study examining 2,340 patients with ND-CKD stage III-V enrolled in three established prospective cohorts of ND-CKD patients under stable care in 40 Italian nephrology clinics previously published [5,8,18]. Study flow chart is illustrated in Fig 1.

SIN-TABLE and RECORD-IT are multicenter studies involving 25 and 19 Italian renal clinics, respectively, while NEPHRO-SUN is a single-center study conducted in the Nephrology Division of Second University of Naples. This Unit served as coordinating center for all studies that had been approved by Institutional Review Board (Second University of Naples). Patients gave written consent to use their data.

The three cohorts were originally built to collect prospective epidemiologic information on consecutive CKD patients under regular care in nephrology clinics. Cohorts shared the main inclusion (established diagnosis of CKD and first visit dating back more than 6 months before baseline) and exclusion criteria (renal replacement therapy, acute kidney injury, active malignancy). After pooling the data, we excluded duplicate subjects and those with CKD stage I-II or with missing values of covariates included in survival analyses (Fig 1).

For the specific purposes of the present study, we stratified patients in two groups: low-proteinuric (LP) group, including patients with basal proteinuria level ≤0.5 g/24h and control group (CON), constituted by those with basal proteinuria >0.5 g/24h. The 0.5 level was chosen according to the new KDIGO classification of CKD that identifies patients with proteinuria above this threshold as having “severe CKD” [10]. We did not select the 0.150 g/24 value to separate the two groups because this threshold, defining normality of protein excretion, may be more appropriate in general population than in our patients with overt CKD, under long-term Nephrology care and treated with antiproteinuric polytherapy.

### Procedures

As for selection criteria, procedures did not differ in the three studies. Participating nephrologists collected anamnestic information, including diagnosis of underlying renal disease, history of cardiovascular (CV) disease, that is, any documented event among myocardial infarction, stroke, angina pectoris, heart failure, peripheral vascular disease. Nephrologists performed the physical examination with assessment of height, body weight, blood pressure (BP), and registered lab results and therapy. Data were collected in anonymous electronic case reports subsequently sent to the coordinating center for analyses.

The classification in two groups was made on the basis of the proteinuria level registered at the first available visit after at least six months of follow up in the Nephrology clinic. That visit represents the baseline visit in the present study.

In the three cohorts, laboratory protocols were standardized with in-house analyses. 24-hour urine collection was obtained to quantify proteinuria and evaluate adherence to the prescribed restriction of protein and salt intake; collection was considered inaccurate, and repeated, if creatinine excretion was outside of the 60 to 140% range of the value calculated according to Dwyer and Kenler [19]. Estimated GFR was calculated by the CKD-EPI equation; as creatinine levels were not standardized to isotope-dilution mass spectrometry values, we reduced levels by 5% according to Skali et al. [20].

### Statistics

Continuous variables are reported as either mean±SD or median and interquartile (IQR) according to their distribution. Comparisons of variables between the two groups are performed by unpaired Student’s t-test or Mann-Whitney test. Categorical variables are analyzed by Chi-square test.

To identify the unmodifiable clinical correlates of LP status, we performed the generalized estimating equation (GEE) regression model, which accounts for cohort cluster effect [21].

In survival analyses, the two endpoints of interest were all-cause death and ESRD, defined as start of chronic dialysis therapy or kidney transplantation. ESRD was reached on the day of the first dialysis session or transplantation; death certificates or hospital records were used to establish date and cause of death. Follow-up expiration date was December 31, 2014. Median follow-up was estimated by the inverse Kaplan-Meier approach. Because ESRD and death before ESRD are competitive events, that is, occurrence of death prevents dialysis therapy initiation or kidney transplantation, we calculated the cumulative incidence of ESRD or death before ESRD using the competing-risk approach and Gray test [22]. Risks of ESRD versus death were compared according to Kochar et al. [23].

Multivariable Cox proportional hazards models were used to estimate hazard ratios (HRs) and 95% confidence intervals (CIs) of the two endpoints. We used Cox models because the cause-specific relative hazard are more appropriate for studying the cause of diseases in the case of a competing event [24].

Models were stratified by cohort (because of the inclusion of three different cohorts) as well as CKD stage (because of the non-linear association between eGFR and ESRD risk), and adjusted for the following baseline covariates, identified *a priori* as risk factors on the basis of previous studies in similar CKD population [2–9]: age, gender, BMI, smoking, diabetes, underlying renal disease, history of CV disease, systolic BP, phosphorus, hemoglobin, proteinuria and use of anti-RAS agents.

A two-tailed P value <0.05 was considered significant. Data were analyzed using SPSS 12.0 (SPSS Inc, Chicago, IL, USA) and R software version 3.1.0 (R Foundation for Statistical Computing, Vienna, Austria).

## Results

### Basal features

Table 1 depicts the basal features of population, overall and stratified by proteinuria category.

**Table 1:** Basal characteristics of patients overall and in the two study groups.

	Overall (n = 2340)	LP (n = 1275)	CON (n = 1065)	P
Age, *years*	67.9±13.3	70.0±12.1	65.4±14.1	<0.001
Male gender, *%*	58.6	55.8	62.0	0.003
Diabetes, *%*	30.6	27.6	34.1	0.001
Cardiovascular disease, *%*	33.7	34.0	33.3	0.719
Body weight, kg	73.4±13.5	73.2±13.4	73.6±13.5	0.550
Body Mass Index, *kg/m*^*2*^	27.7±5.0	27.8±5.0	27.7±4.9	0.619
Current smoking, *%*	11.3	10.4	12.5	0.105
Nephrology care, *months*	15 [12–22]	14 [12–22]	15 [11–22]	0.507
Blood Pressure, *mmHg*	138±18/79±11	137±18/78±11	140±18/80±11	<0.001/<0.001
eGFR, *mL/min/1*.*73 m*^*2*^	31.2±14.0	34.8±13.5	26.8±13.2	<0.001
24h Proteinuria, *g*	0.43 [0.13–1.09]	0.15 [0.06–0.28]	1.20 [0.80–2.07]	-
24h Urinary sodium, *mmol*	148±63	143±62	155±64	<0.001
Primary renal disease				<0.001
HTN	31.8	39.8	22.1	
DN	14.7	11.3	18.8	
GN	14.1	7.8	21.6	
PKD	4.5	5.0	3.8	
TIN	8.3	7.8	9.0	
Other/Unknown	26.7	28.3	24.7	
Calcium, *mg/dL*	9.3±0.6	9.4±0.6	9.2±0.7	<0.001
Phosphorus, *mg/dL*	3.8±0.8	3.7±0.8	3.9±0.8	<0.001
sAlbumin, *g/dL*	4.0±0.5	4.1±0.5	3.9±0.5	<0.001
Hemoglobin, *g/dL*	12.5±1.7	12.7±1.7	12.3±1.7	<0.001
Triglycerides, *mg/dL*	126 [93–172]	119 [89–161]	135 [97–190]	<0.001
Uric acid, *mg/dL*	6.2±1.7	6.2±1.7	6.3±1.7	0.503
LDL-Cholesterol, *mg/dL*	108±33	107±32	110±33	0.015
BP lowering drugs, *n/patient*	2.5±1.3	2.4±1.2	2.5±1.3	0.201
Anti-RAS use, *% patients*	75.3	76.8	73.5	0.068

Values are means (SD), or median (interquartile range), or percentages. LP, proteinuria ≤0.5 g/24h; CON, proteinuria >0.5 g/24h. HTN, hypertensive nephropathy; DN, diabetic nephropathy; GN, glomerulonephritis; TIN, tubulointerstitial nephropathy; PKD, polycystic kidney disease; eGFR, GFR estimated by the CKD-EPI equation; sAlbumin, serum albumin.

Whole cohort was characterized by a high-risk profile, as testified by the high prevalence of diabetes, CV disease, and advanced CKD (eGFR <30 mL/min/1.73 m^2^ in 48.3%). LP patients constituted 54.5% of whole population. In this group, as compared with CON, mean age was almost 5 years higher while male gender, smoking habit, diabetic and glomerular disease were less frequent; BMI and prior nephrology care were similar. Mean eGFR was 8 mL/min higher in LP vs CON, with CKD stage 3 being more frequent in the former group (62.2% vs 37.1%); accordingly, also hemoglobin was higher despite lower use of epoietin (16.4% vs 21.4% in LP and CON, respectively, P = 0.001), while systolic BP was lower in the presence of similar antihypertensive treatment. Metabolic abnormalities were also less severe in LP than CON; in particular, serum albumin and calcium were higher whereas lipid profile was better controlled. Serum phosphorus (P) was normal in the vast majority of cohort (P≤4.5 mg/dL in 85% population), however P levels were lower in LP with greater prevalence of normal values in this group (89%) than in CON (81%) despite a lower use of P binders (7.5% in LP and 10.8% in CON, P = 0.004).

Clinical correlates of LP status at GEE analysis were older age, female gender, non-diabetic status, absence of diabetic, glomerular or tubulointerstitial nephropathy, and higher GFR (Table 2).

**Table 2:** Multivariable Cox models of determinants of ESRD and all-cause death in LP patients.

	ESRD			Death		
	HR	95% CI	P	HR	95% CI	P
Age (for 1 year)	**0.97**	**0.96–0.99**	**<0.001**	**1.09**	**1.07–1.11**	**<0.001**
Male gender	1.36	0.96–1.92	0.08	1.18	0.85–1.64	0.31
Body Mass Index (kg/m^2^)	**0.96**	**0.92–1.00**	**0.03**	1.00	0.96–1.03	0.84
Diabetes	1.48	0.88–2.49	0.14	1.22	0.81–1.85	0.35
Cardiovascular disease	1.13	0.76–1.67	0.54	1.21	0.89–1.63	0.23
Smoking	1.40	0.86–2.27	0.17	**1.77**	**1.13–2.77**	**0.01**
HTN	Ref	Ref	Ref	Ref	Ref	Ref
DN	0.73	0.34–1.58	0.43	1.04	0.61–1.77	0.89
GN	1.28	0.63–2.58	0.50	0.83	0.37–1.85	0.64
PKD	**2.98**	**1.73–5.15**	**<0.001**	1.21	0.54–2.71	0.65
TIN	1.33	0.71–2.49	0.37	1.39	0.80–2.40	0.24
Other/Unknown	1.10	0.70–1.72	0.69	0.77	0.52–1.15	0.20
Hemoglobin (g/dL)	0.90	0.80–1.02	0.09	**0.88**	**0.79–0.98**	**0.02**
Systolic BP (5 mmHg)	1.02	0.97–1.07	0.40	1.01	0.97–1.06	0.50
Anti-RAS	0.88	0.61–1.28	0.51	0.88	0.63–1.24	0.47
Phosphate (mg/dl)	**1.35**	**1.09–1.67**	**0.01**	1.10	0.92–1.32	0.29
24h Proteinuria (g/24h)	2.83	0.95–8.42	0.06	**3.00**	**1.09–8.21**	**0.03**

Analyses were stratified by cohort and CKD stage. HTN, hypertensive nephropathy; DN, diabetic nephropathy; GN, glomerulonephritis; TIN, tubulointerstitial nephropathy; PKD, polycystic kidney disease BP, blood pressure; RAS, renin angiotensin system.

### Survival analyses

**Fig 2:**
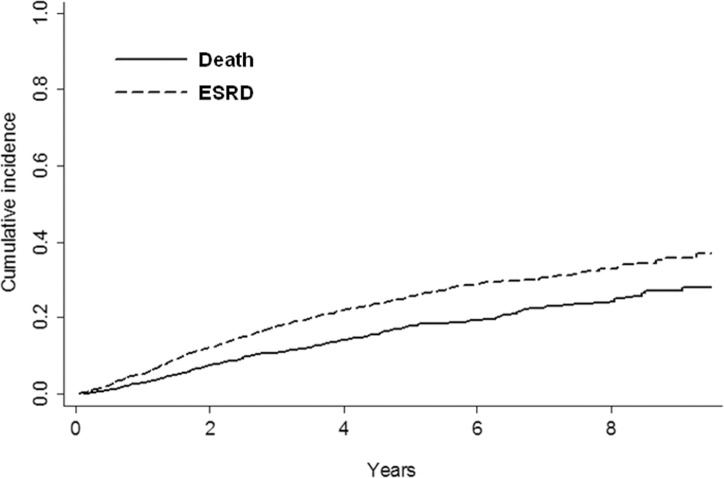
Cumulative incidence probability of ESRD and all-cause death before ESRD, by competing risk analysis, in the whole study population (n = 2340). P <0.0001.

In the whole population, risk of ESRD overcame mortality risk (Fig 2), with specific determinants being remarkably different (Table 3).

**Table 3:** Multivariable Cox models of determinants of ESRD and all-cause death in the whole study population (n = 2340).

	ESRD			Death		
	HR	95% CI	P	HR	95% CI	P
Age (for 1 year)	**0.98**	**0.97–0.99**	**<0.001**	**1.08**	**1.07–1.09**	**<0.001**
Male gender	**1.34**	**1.11–1.60**	**0.01**	**1.38**	**1.10–1.73**	**0.01**
Body Mass Index (kg/m^2^)	**0.98**	**0.96–1.00**	**0.02**	0.99	0.97–1.01	0.39
Diabetes	1.02	0.79–1.31	0.90	**1.33**	**1.01–1.75**	**0.04**
Cardiovascular disease	**1.28**	**1.06–1.55**	**0.01**	**1.31**	**1.06–1.61**	**0.01**
Smoking	1.14	0.88–1.47	0.32	**1.41**	**1.02–1.95**	**0.04**
HTN	Ref	Ref	Ref	Ref	Ref	Ref
DN	1.03	0.73–1.46	0.87	1.19	0.84–1.69	0.33
GN	1.27	0.96–1.69	0.10	1.10	0.74–1.65	0.64
PKD	**2.16**	**1.53–3.05**	**<0.001**	0.78	0.38–1.61	0.51
TIN	1.01	0.71–1.42	0.98	1.31	0.88–1.94	0.18
Other/Unknown	0.95	0.74–1.23	0.71	1.00	0.75–1.33	0.99
Hemoglobin (g/dL)	**0.90**	**0.85–0.96**	**<0.001**	**0.89**	**0.83–0.96**	**0.01**
Systolic BP (5 mmHg)	1.02	1.00–1.05	0.07	1.00	0.97–1.03	0.88
Anti-RAS	**0.79**	**0.65–0.96**	**0.02**	**0.78**	**0.62–0.98**	**0.04**
Phosphate (mg/dl)	**1.27**	**1.14–1.40**	**<0.001**	1.04	0.90–1.19	0.62
24h Proteinuria (g/24h)	**1.14**	**1.09–1.18**	**<0.001**	**1.12**	**1.05–1.20**	**0.01**

Analyses were stratified by cohort and CKD stage. HTN, hypertensive nephropathy; DN, diabetic nephropathy; GN, glomerulonephritis; TIN, tubulointerstitial nephropathy; PKD, polycystic kidney disease BP, blood pressure; RAS, renin angiotensin system.

When the two groups were examined separately, different results emerged in the presence of similar follow up (median follow-up was 48.5 months, IQR 38.8–66.2, in LP and 48.5, IQR 37.5–62.8, in CON).

Specifically, ESRD was less frequent in LP as it occurred in 156 LP and 418 CON, with incidence rate of 2.7 and 11.7/100 pt-y, respectively. A minor difference was detected in mortality; all-cause death was in fact reported in 212 LP and 200 CON, with incidence rate of 3.7 and 5.6/100 pt-y, respectively.

**Fig 3:**
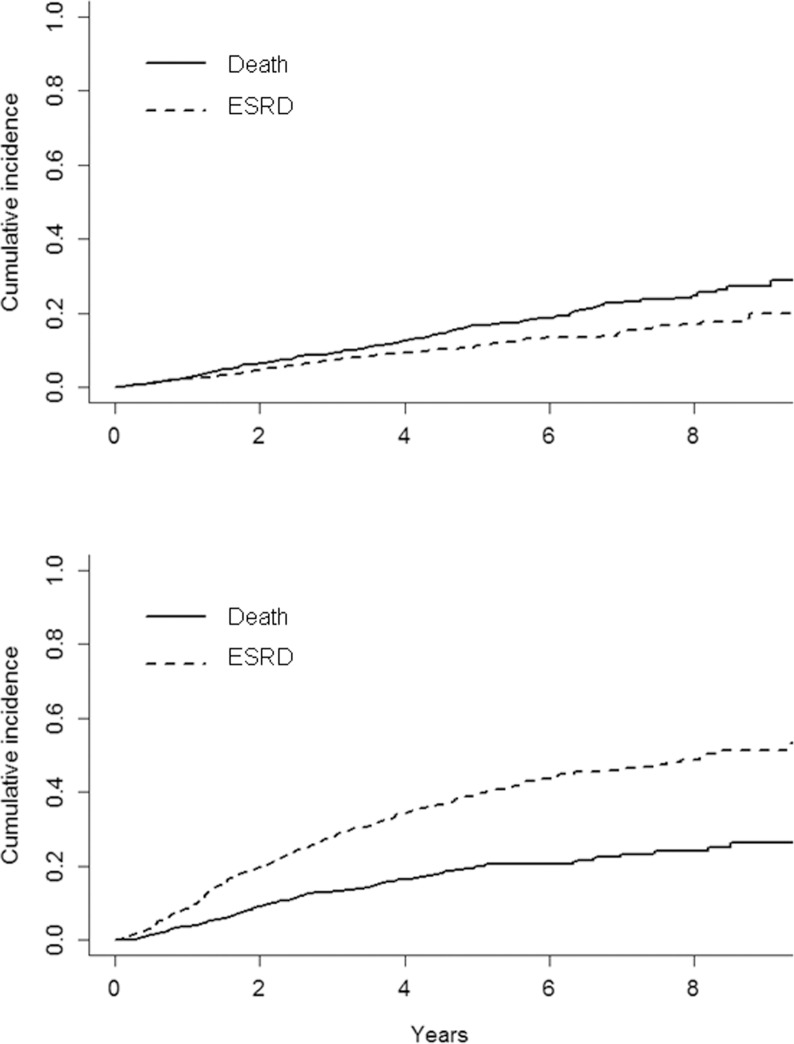
Cumulative incidence probability of ESRD and all-cause death before ESRD, by competing risk analysis, in LP (top) and CON (bottom) patients. P values were <0.0001 and 0.002 in LP and CON, respectively.

Competing risk analysis in the two groups is depicted in Fig 3.

Progression to dialytic stage remarkably overcame mortality in CON (P<0.0001) while LP patients were characterized by a greater risk of all-cause death than ESRD (P = 0.002). When analysis of prognosis in LP group was limited to stage 3 only, we observed mortality rates similar to that of the whole LP group and a renal risk that was only slightly lower (data not shown).

Conversely, CV risk (fatal and non-fatal CV events) by competing risk analysis resulted similar in the two groups (data not shown).

Major differences emerged in the modifiable risk factors (that is, those that are targets of therapy) of ESRD and all-cause death. In LP, higher P and lower BMI were main determinants of ESRD, while proteinuria, smoking and lower hemoglobin predicted mortality (Table 4).

**Table 4:** Multivariable Cox models of determinants of ESRD and all-cause death in LP patients.

	ESRD			Death		
	HR	95% CI	P	HR	95% CI	P
Age (for 1 year)	**0.97**	**0.96–0.99**	**<0.001**	**1.09**	**1.07–1.11**	**<0.001**
Male gender	1.36	0.96–1.92	0.08	1.18	0.85–1.64	0.31
Body Mass Index (kg/m^2^)	**0.96**	**0.92–1.00**	**0.03**	1.00	0.96–1.03	0.84
Diabetes	1.48	0.88–2.49	0.14	1.22	0.81–1.85	0.35
Cardiovascular disease	1.13	0.76–1.67	0.54	1.21	0.89–1.63	0.23
Smoking	1.40	0.86–2.27	0.17	**1.77**	**1.13–2.77**	**0.01**
HTN	Ref	Ref	Ref	Ref	Ref	Ref
DN	0.73	0.34–1.58	0.43	1.04	0.61–1.77	0.89
GN	1.28	0.63–2.58	0.50	0.83	0.37–1.85	0.64
PKD	**2.98**	**1.73–5.15**	**<0.001**	1.21	0.54–2.71	0.65
TIN	1.33	0.71–2.49	0.37	1.39	0.80–2.40	0.24
Other/Unknown	1.10	0.70–1.72	0.69	0.77	0.52–1.15	0.20
Hemoglobin (g/dL)	0.90	0.80–1.02	0.09	**0.88**	**0.79–0.98**	**0.02**
Systolic BP (5 mmHg)	1.02	0.97–1.07	0.40	1.01	0.97–1.06	0.50
Anti-RAS	0.88	0.61–1.28	0.51	0.88	0.63–1.24	0.47
Phosphate (mg/dl)	**1.35**	**1.09–1.67**	**0.01**	1.10	0.92–1.32	0.29
24h Proteinuria (g/24h)	2.83	0.95–8.42	0.06	**3.00**	**1.09–8.21**	**0.03**

Analyses were stratified by cohort and CKD stage. HTN, hypertensive nephropathy; DN, diabetic nephropathy; GN, glomerulonephritis; TIN, tubulointerstitial nephropathy; PKD, polycystic kidney disease BP, blood pressure; RAS, renin angiotensin system.

In CON, while lower hemoglobin was associated with either outcome, proteinuria predicted only ESRD (Table 5).

**Table 5:** Multivariable Cox models of determinants of ESRD and all-cause death in CON patients.

	ESRD			Death		
	HR	95% CI	P	HR	95% CI	P
Age (for 1 year)	**0.99**	**0.98–1.00**	**0.01**	**1.08**	**1.07–1.10**	**<0.001**
Male gender	**1.25**	**1.01–1.55**	**0.04**	**1.45**	**1.03–2.04**	**0.03**
Body Mass Index (kg/m^2^)	0.98	0.96–1.00	0.11	0.99	0.96–1.03	0.71
Diabetes	0.89	0.66–1.19	0.43	1.38	0.94–2.03	0.10
Cardiovascular disease	**1.39**	**1.11–1.75**	**0.01**	**1.64**	**1.21–2.22**	**0.01**
Smoking	1.01	0.74–1.37	0.97	1.16	0.71–1.89	0.57
HTN	Ref	Ref	Ref	Ref	Ref	Ref
DN	0.98	0.65–1.47	0.92	1.23	0.74–2.05	0.42
GN	1.07	0.77–1.48	0.70	1.06	0.64–1.77	0.81
PKD	**1.70**	**1.05–2.78**	**0.03**	0.18	0.02–1.30	0.09
TIN	0.81	0.53–1.25	0.35	1.29	0.72–2.32	0.39
Other/Unknown	0.92	0.67–1.25	0.58	1.26	0.83–1.92	0.28
Hemoglobin (g/dL)	**0.91**	**0.85–0.97**	**0.01**	**0.89**	**0.81–0.98**	**0.02**
Systolic BP (5 mmHg)	1.02	0.99–1.05	0.19	0.97	0.92–1.01	0.16
Anti-RAS	**0.74**	**0.58–0.94**	**0.01**	**0.69**	**0.50–0.96**	**0.03**
Phosphate (mg/dl)	**1.26**	**1.12–1.42**	**<0.001**	0.89	0.72–1.11	0.32
24h Proteinuria (g/24h)	**1.11**	**1.07–1.17**	**<0.001**	1.05	0.95–1.15	0.34

Analyses were stratified by cohort and CKD stage. HTN, hypertensive nephropathy; DN, diabetic nephropathy; GN, glomerulonephritis; TIN, tubulointerstitial nephropathy; PKD, polycystic kidney disease BP, blood pressure; RAS, renin angiotensin system.

**Fig 4:**
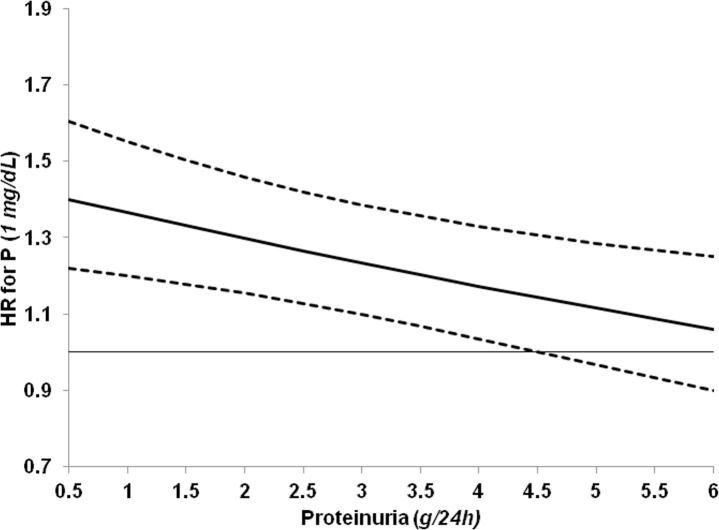
Adjusted hazard ratios (solid line) and 95% confidence intervals (dashed lines) of one-unit increase of serum phosphorus (P) by 24h proteinuria in the prediction of ESRD in control patients. The horizontal line represents hazard ratio 1. Beta value of the interaction P*Proteinuria is -0.050 (P 0.004). Hazards are stratified by cohort and CKD stage and adjusted for all covariates in Cox model reported in Table 5.

In CON group, the increase of P levels also had a worsening effect on renal prognosis with 1 mg/dL increase being linked to 26% higher risk of ESRD. To gain more insights into this association in CON group, characterized by a wider range of proteinuria levels versus LP, we tested the presence of interaction between P and proteinuria on ESRD risk. When the interaction term P*proteinuria was added to the Cox model on ESRD (Table 5), we found a negative interaction between proteinuria and P (Beta = -0.050, P = 0.004), that is, the renal risk related to P levels decreased in the presence of higher proteinuria (Fig 4). In CON, this interaction on ESRD risk persisted (beta = -0.0469, P = 0.004) when eGFR was included as covariate, thus replacing stratification by CKD stage. As observed in CON, moreover, the interaction P*proteinuria was still significant when added to the Cox models in the whole population (Table 3) for ESRD risk (beta = -0.066 P<0.001) but not for mortality (beta = -0.060 P = 0.172).

## Discussion

Information accrued over the past decade indicate that nephrology care is associated with better CKD patients’ prognosis [25–28]. However, efforts aimed at further improving risk stratification and management are still mandatory now due to the progressively expanding population of CKD patients followed in renal clinics, [1]. This study adds novel information on this critical issue by showing that in a large population of referred CKD patients, low proteinuria status (<0.5 g/24h) conveys *per se* a unique risk profile in terms of adverse outcome and risk factors.

We found that LP patients constituted more than half of the examined population. Female gender, older age, nondiabetic status, and higher GFR were all correlates of this condition; as expected, moreover, hypertensive nephropathy was the most frequent primary disease in this large subgroup of patients. Few studies have evaluated low-proteinuric CKD, and exclusively in terms of cross-sectional analyses aimed at estimating prevalence rates of this condition. In the U.S. general population with eGFR <60 mL/min/1.73m^2^, normoalbuminuria was observed in 66% cases, specifically in 56% diabetic and 77% nondiabetic CKD patients [13]. A high prevalence of low albuminuria (72%) was also reported in a cross-sectional analysis of the German Chronic Kidney Disease cohort, including 5,217 patients with mild renal impairment (eGFR 47±17 mL/min) [16]. The higher prevalence of low proteinuria in these two previous cross-sectional studies versus our study likely relates to the milder degree of renal disease of those cohorts; in those studies, in fact, mean eGFR was approximately 15 ml higher than in our population. Indeed, a positive association between severity of GFR impairment and proteinuria levels is a consistent observation in ND-CKD population [2–6].

Besides and beyond the differing basal profile, LP remarkably differed from CON in their long-term prognosis. To better analyze risk status, we used the competing risk approach, that is, the survival analysis recommended when a patient is at risk of more than one type of event, as in the case of ND-CKD where premature mortality can significantly hinder the true incidence of ESRD [10]. At variance with CON group, LP patients showed a higher risk of death than ESRD (Fig 2). This is an “inverse” fate when considering the study setting. Indeed, when patients under nephrology care are examined as whole population, the dominant clinical outcome is ESRD rather than death [2–6]. Similarly, a higher competing risk of ESRD versus death becomes evident in cohorts of patients selected for trials aimed at slowing CKD progression [29,30]. The dominant outcome of LP patients possibly depends on clustering of factors contributing to a relatively low ESRD risk. In this regard, besides lower proteinuria, female gender, older age, higher prevalence of hypertensive nephropathy and more preserved eGFR may all act as main additional modifiers of prognosis as they portend a higher risk of death versus ESRD [3,5,6,8,11].

Defining CKD is matter of debate in the current nephrology literature, especially in terms of the threshold value of eGFR used to make the diagnosis of CKD stage 3 [31–33]. In LP group, CKD stage 3 was prevalent; however, when analyzing prognosis of stage 3 only, mortality was not different while renal risk was only slightly lower as compared to the whole LP group (data not shown). This observation may be dependent on the specific study setting, that is, inclusion of patients that likely have “true” CKD, as suggested by the prolonged and continuous nephrology follow up. The importance of the clinical setting is supported by the comparison of prognosis in our study versus early work in general population and/or unreferred cohorts. Specifically, the residual risk of progression to ESRD in LP, though relatively lower when compared to what observed for HP patients, was still relevant in absolute terms. In large studies in general population [34,35], in fact, the mean incidence rate of ESRD ranged from 0.004 to 0.1/100 subject-y, a 30- to 700-fold lower rate than what recorded in LP patients (2.7/100 pt-y). Renal risk of LP patients still remains substantially greater when other populations at high risk for ESRD are examined. Indeed, ESRD rates among patients with severe hypertension (BP >160/90 mmHg) or severe obesity did not exceed 0.020/100 and 0.3/100 pt-y, respectively [34–36]. Furthermore, the reported ESRD incidence in patients with type 2 diabetic nephropathy and low proteinuria (ACR ≤1 g/g), enrolled in IDNT and RENAAL trials [37], was still lower (1.9/100 pt-y) than that observed in LP patients. Finally, CKD patients exclusively followed in the primary care setting, and with age and eGFR similar to our referred LP group [38], had a 10-fold lower ESRD incidence (0.25/100 pt-y). Overall, these data therefore indicate that CKD patients are at a relatively higher risk of ESRD even after a prolonged nephrology care and even in the presence of low proteinuria.

Interestingly, the two groups showed similar CV risk (fatal and non-fatal CV events). Reason for this result is not readily apparent; however, it may depend on the fact that the higher CV risk conveyed by older age in LP is counterbalanced in CON by the larger prevalence of diabetes, besides and beyond the higher proteinuria in this group that per se increases CV risk [39].

Noteworthy, the separate analysis of the two groups also allows to optimize risk profile and discrimination of the risk factors specific to either outcome. Due to the clinical setting of tertiary nephrology care, focusing on risk factors potentially modifiable by therapy becomes particularly important. When examining mortality risk, besides low hemoglobin, that had a significant predictive role in either group, smoking emerged as a major modifiable determinant specific to LP condition. LP patients may be therefore more exposed than CON to the worsening effect of smoking on survival. It is reasonable to hypothesize that this association may relate to the superimposition of smoking, that *per se* is linked to atheromatosis and vascular calcification, over an ischemic background correlated to the older age and the higher prevalence of hypertensive disease in this group [40–42]. The remarkable prognostic role of this habit suggests that more time and efforts should be dedicated particularly in these patients for counseling on smoking cessation. Similar to smoking, also the degree of proteinuria predicted mortality in LP only; this finding may be coherent to the role of low-grade proteinuria as recognized proxy of atherosclerosis-associated vasculopathy [43].

As observed for mortality risk, the modifiable determinants of renal prognosis differed in LP. Higher BMI heralded a lower ESRD risk only in this group. It is possible that the renal protective effects of “better” nutritional reserves may be enhanced in LP due to their distinctive characteristics, older age and a pro-atherosclerotic background *in primis* [44]. Indeed, a recent large study in general population has shown that moderately increased BMI -prevalent feature in our population- protects against loss of renal function in older patients [45].

An original finding, of great clinical relevance, is the linkage between phosphorus and proteinuria on renal prognosis. Phosphorus emerged as the main modifiable determinant of ESRD in LP. This role persisted also in CON patients although the association of phosphorus and risk of ESRD was significantly attenuated in the presence of higher levels of proteinuria (Fig 3). Relevance of these observations increases when considering that phosphorus levels, normal in the vast majority of cohort, were significantly lower in LP. This difference is mainly due to the higher eGFR in LP; however, we cannot exclude the contribution of a lower tubular reabsorption of phosphorus linked to the low proteinuria in this group, as recently suggested by an experimental study [46].

The interaction between phosphorus and proteinuria on renal risk adds novel insights into the critical -and so far still unsolved- issue of the definition of the optimal phosphorus levels in ND-CKD [47,48]. Indeed, while previous studies have collectively evidenced that P levels, even in the normal range, predict progression to ESRD [49], data obtained in more than 10,000 CKD patients with eGFR <60 ml/min per 1.73m^2^ confute this independent association [50]. Our results suggest that the predictive role of P on renal outcome is strongly influenced by the entity of proteinuria. In this regard, two hypotheses can be made. The first is merely related to the potential limitations intrinsic to all survival analyses, that is, the strength of an association between exposure to a given risk factor (*proteinuria*) and outcome (*ESRD*) may be so high to attenuate the role of other risk factors (*phosphorus*). The second is more based on pathophysiology; indeed, the causative role of phosphorus on CKD progression, which is mainly mediated by tubulo-interstitial fibrosis similarly to proteinuria [51,52], may be greater if the proteinuria-induced renal injury is less evident. This latter hypothesis is supported by the evidence that the nephroprotective effects of antiproteinuric therapy increases when phosphorus levels are low [53,54].

Interestingly, the two groups shared a higher ESRD risk in PKD versus other diagnosis of primary renal disease. This observation suggests that, at least so far, this specific renal disease suffers of the paucity of effective therapeutic tools with respect to other renal diseases. Indeed, in a recent study by our group, we found that after intensification of therapy during first year of Nephrology care in CKD patients, risk of ESRD decreased in all renal diseases but PKD [8].

Our study is limited by the assessment of predictors only at baseline; nevertheless, the prolonged follow up in nephrology prior to basal visit—≥12 months on average—reasonably excludes substantial changes of risk factors in the subsequent period. Furthermore, our analysis does not allow to distinguish patients who reverted from high proteinuria status from those with proteinuria persistently low; however, the study was designed to evaluate LP status *per se* with the aim of refining risk stratification after long-standing nephrology care. Finally, analyses of factors associated with ESRD and death did not account for PTH levels as this measure was missing in most patients. On the other hand, the study has strengths such as the size of population, which is relatively large when considering the referral status of patients, as well as the fact that survival analyses were adjusted for several factors, including the renal diagnoses which in many population based cohorts is not the case.

## Conclusions

This study provides novel information on the ND-CKD population under regular nephrology care. We found that LP patients are the majority and show distinctive basal features. More important, they are more exposed to death than ESRD and do present differences in the modifiable determinants of either outcome; indeed, while smoking plays a role for mortality, lower BMI and higher phosphorus levels -even if in the normal range- are predictors of ESRD. These data extend to the population of patients regularly followed in nephrology the clinical relevance of the 0.5 g/24h proteinuria threshold, that has been indicated by the new KDIGO guidelines as a simple marker to stratify the risk in the general ND-CKD population [10]. Overall, these data support the need to further study the low proteinuric CKD population to guide management.

## Supporting information

S1 Dataset Complete dataset. (SAV)
